# Radiosynthesis of Stable ^198^Au-Nanoparticles by Neutron Activation of α_v_β_3_-Specific AuNPs for Therapy of Tumor Angiogenesis

**DOI:** 10.3390/ph16121670

**Published:** 2023-11-30

**Authors:** Güllü Davarci, Carmen Wängler, Klaus Eberhardt, Christopher Geppert, Ralf Schirrmacher, Robert Freudenberg, Marc Pretze, Björn Wängler

**Affiliations:** 1 Molecular Imaging and Radiochemistry, Clinic of Radiology and Nuclear Medicine, Medical Faculty Mannheim of Heidelberg University, 68167 Mannheim, Germany; guellue.davarci@medma.uni-heidelberg.de; 2 Biomedical Chemistry, Clinic of Radiology and Nuclear Medicine, Medical Faculty Mannheim of Heidelberg University, 68167 Mannheim, Germany; carmen.waengler@medma.uni-heidelberg.de; 3 Mannheim Institute for Intelligent Systems in Medicine MIISM, Medical Faculty Mannheim of Heidelberg University, 68167 Mannheim, Germany; 4 Research Reactor TRIGA Mainz, Institute for Nuclear Chemistry, Johannes-Gutenberg-Universität Mainz, 55128 Mainz, Germany; eberha@uni-mainz.de (K.E.); cgeppert@uni-mainz.de (C.G.); 5 Department of Oncology, Division of Oncological Imaging, University of Alberta, Edmonton, AB T6G 2R3, Canada; schirrma@ualberta.ca; 6 Department of Nuclear Medicine, University Hospital Carl Gustav Carus, TU Dresden, 01307 Dresden, Germany; robert.freudenberg@ukdd.de

**Keywords:** gold nanoparticles, [^198^Au]AuNPs, radioactive, tumor therapy, tumor angiogenesis, RGD peptide

## Abstract

This paper reports on the development of stable tumor-specific gold nanoparticles (AuNPs) activated by neutron irradiation as a therapeutic option for the treatment of cancer with high tumor angiogenesis. The AuNPs were designed with different mono- or dithiol-ligands and decorated with different amounts of Arg-Gly-Asp (RGD) peptides as a tumor-targeting vector for α_v_β_3_ integrin, which is overexpressed in tissues with high tumor angiogenesis. The AuNPs were evaluated for avidity in vitro and showed favorable properties with respect to tumor cell accumulation. Furthermore, the therapeutic properties of the [^198^Au]AuNPs were evaluated in vitro on U87MG cells in terms of cell survival, suggesting that these [^198^Au]AuNPs are a useful basis for future therapeutic concepts.

## 1. Introduction

In recent years, gold nanoparticles (AuNPs) have received serious attention since their first use as radioactive ^198^Au-nanocolloids for nanobrachytherapy in the early 1950s [1,2]. The synthesis of ultra-small AuNPs (<5 nm) [3] with multimerization of target-specific effectors on their surface leads to a new form of targeted AuNPs with higher target avidity compared to the effectors only [4]. The combination of the target-specific accumulation and a phenomenon typically known as the “enhanced permeability and retention” (EPR) effect [5], leads to a higher tumor accumulation [6]. Therefore, AuNPs with a higher renal clearance [7] for theranostic purposes [8,9,10] were developed in recent years, equipped with small molecules [11], peptides [12], near-infrared dyes [13,14], and radionuclides [15,16,17,18,19]. PEGylation of the AuNPs leads to a higher bioavailability as it prevents the formation of a protein corona around the AuNPs in vivo [20,21]. The high affinity of sulfur for gold surfaces and the formation of stable and covalent Au-S bonds [22] allows a fast and easy functionalization of AuNPs with (di-)thiol-modified (bio)molecules [23]. In addition, the use of dithiols as a surface binding motif leads to a higher stability of the AuNPs [24]. Of particular interest is their therapeutic application [25], especially their ability to be used as radiosensitizers by Auger–Meitner electron emission induced by gamma activation [26,27,28] or by direct neutron activation of natural ^197^AuNPs generating [^198^Au]AuNPs (t_1/2_ = 2.69 d, β^−^_max_ 961 keV, 98.99%; γ 412 keV, 95.62%) [12,29,30,31,32].

The focus of this work was the development of highly stable targeted gold nanoparticles for neutron activation [33]. Therefore, AuNPs with mono- and di-thiol linkers with low and high loading of target-specific peptides were synthesized to compare their specific avidity in cell binding assays and their stability during and after neutron irradiation. To achieve target-specific accumulation in tissues with high tumor angiogenesis, the AuNPs were functionalized with a c(RGDfK) derivative [32,34]. The Arg-Gly-Asp (RGD) peptide motif is known to bind to the transmembrane α_v_β_3_ integrin [35,36], which is overexpressed in tumor angiogenesis in tumors of various origins, for example, on glioma cells (U87MG) [37,38,39].

## 2. Results

### 2.1. Synthesis and Functionalization of Gold Nanoparticles

**Figure 1:**
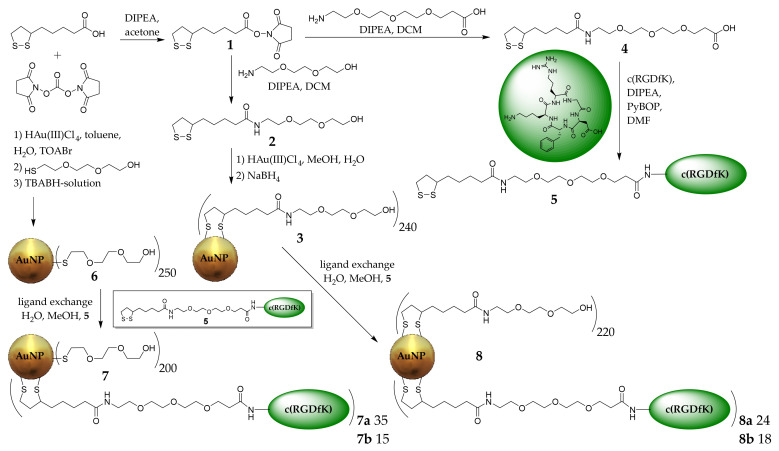
Synthesis of the different RGD-functionalized AuNPs **7a**, **7b**, **8a** and **8b**. The synthesis of c(RGDfK) is described in Appendix A.

Integrin α_v_β_3_, a transmembrane protein expressed on endothelial cells, binds the RGD triple amino acid peptide motif of extracellular matrix proteins. Growing malignant tumors require continuous angiogenesis, and the integrin α_v_β_3_ is overexpressed for this purpose. As a result, α_v_β_3_ is preferentially expressed in tumor angiogenesis and is a potential target for AuNPs decorated with RGD peptides [36]. Therefore, ultra-small AuNPs **3** and **6** (3 ± 2 nm) were synthesized by Turcu et al. [40] and Brust and Schiffrin [41], respectively. The AuNPs contained thiol-PEG_3_-OH or a thioctic acid(TA)-PEG_3_-OH derivative **2** used as the stabilizing ligands and to achieve enhanced biocompatibility (Figure 1). The AuNPs were further functionalized by ligand exchange with low and high amounts (4–8 mg) of TA-PEG_4_-c(RGDfK) derivative **5** to obtain mixed AuNP-thio-PEG-dithio-PEG-RGD **7a** (high RGD loading), **7b** (low RGD loading) and AuNP-dithio-RGD **8a** (high RGD loading), and **8b** (low RGD loading), respectively. The AuNPs were purified by dialysis and size-exclusion chromatography. The size and stability of AuNPs **7a**,**b**, and **8a**,**b** were confirmed by UV/Vis spectroscopy and high performance liquid chromatography (HPLC).

**Figure A1:**
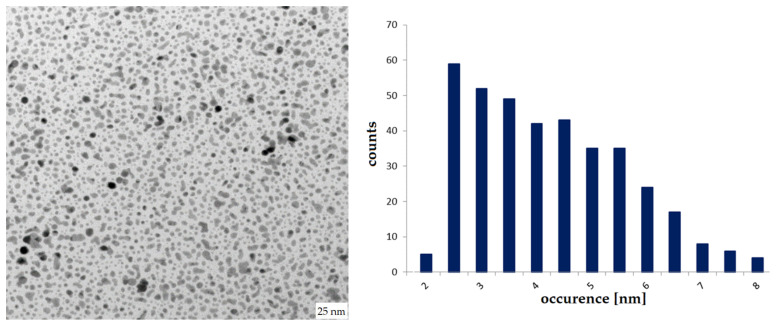
Transmission electron microscope image of AuNP **6** and corresponding histogram of AuNP diameter distribution.

**Figure A2:**
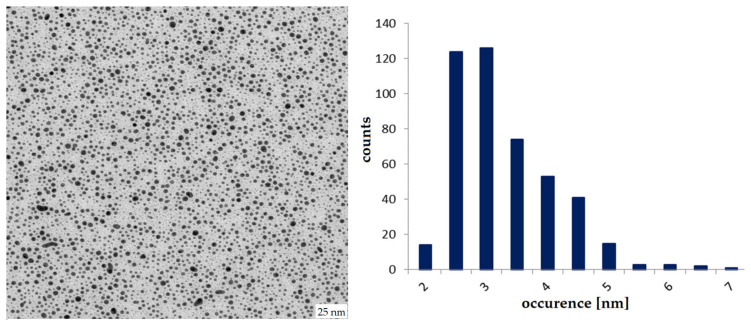
Transmission electron microscope image of AuNP **3** and corresponding histogram of AuNP diameter distribution.

The organic shell of the AuNPs was characterized by mass loss using thermogravimetric analyses for each functionalization step. After knowing the number of newly attached molecules, a formula by Zhu et al. was used to calculate the total molar mass of the AuNPs [42] (Table 1). A brief description of the synthesis and characterization can be found in Appendix A. All AuNPs were fully characterized by thermogravimetric analysis (TGA) (Table 1), electron microscopy (EM) (Figure A1 and Figure A2), UV/Vis spectroscopy (Figure A9), HPLC (Figure A10), and nuclear magnetic resonance spectroscopy (NMR) (Figure A11, Figure A12, Figure A13, Figure A14, Figure A15 and Figure A16). The AuNPs could be stored in lyophilized form at −20 °C for >12 months without loss of integrity. In contrast, when stored in solution at room temperature, aggregation in the form of precipitation could occur within weeks, especially for peptide-decorated particles [43].

### 2.2. Neutron Irradiation Experiments

**Figure 2:**
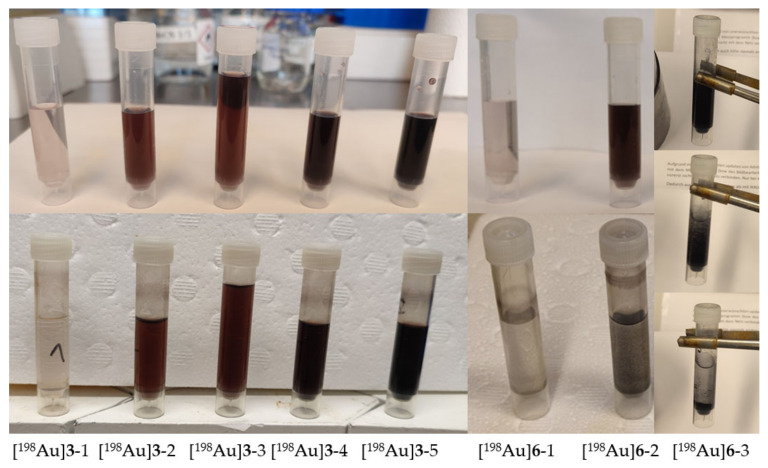
Different amounts of AuNPs before (**top**) and after (**bottom**) neutron activation. [^198^Au]**6**-3 (**right**) shows a suspension immediately after irradiation (**top**), followed by precipitation within 0.5 min (**middle**) and 1 min (**bottom**).

**Figure 3:**
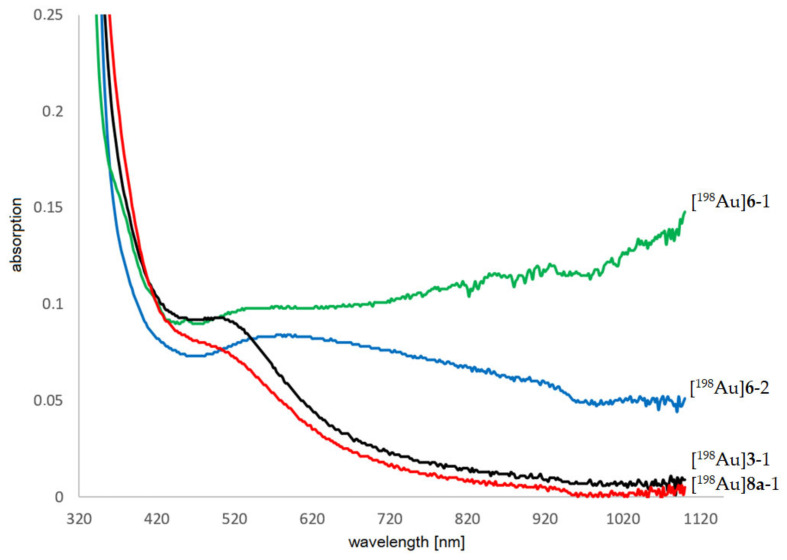
UV-Vis spectra of neutron-activated AuNPs. Intact [^198^Au]**3**-1 (black line, 25 µg/mL, irradiation 60 min) and [^198^Au]**8a**-1 (red line, 25 µg/mL, irradiation 60 min). Particle aggregation can be seen as broadening of the typical plasmon band at 514 nm for [^198^Au]**6**-1 (green line, 25 µg/mL, irradiation 15 min) and [^198^Au]**6**-2 (blue line, 25 µg/mL, irradiation 60 min).

First neutron irradiation experiments with thermal neutrons at the TRIGA Mainz reactor were performed with non-tumor specific AuNP-dithio-PEG **3** (**3**-1–**3**-5) and AuNP-thio-PEG **6** (**6**-1–**6**-3) in different weights and concentrations. Samples were frozen and removed from the freezer immediately before irradiation. Irradiation was performed at 100 kW for 1–2 h with a neutron flux of 1.6 × 10^12^ cm^−2^ × s^−1^. With the reactor running, the background dose rate (DR) at the measurement position was ~2 µSv/h. Gamma measurements were not possible for probes >500 µg on the day of irradiation due to the high activity. The dead time for samples **3**-5 (Table 2) was about 30 min at the end of the bombardment and still 7.5 min at 20 cm distance. Therefore, most of the gamma measurements of [^198^Au]AuNPs had to be performed one day after irradiation. Sample [^198^Au]**3**-5 still had ~3% dead time in 20 cm distance (Table 2). Precipitation was observed for [^198^Au]**6** but not for [^198^Au]**3** in the solution or on the vessel wall in any case (Figure 2). The activated samples were stored in the freezer for transport and further experiments. In addition, the half-life of [^198^Au]**3** was determined experimentally (mean 2.80 ± 0.07 d) by measuring the activity of different concentrations with a gamma counter for 28 d (Figure A8). UV-Vis measurements showed a strong broadening of the plasmon bands for [^198^Au]**6**-1 and [^198^Au]**6**-2, indicating aggregation (Figure 3). For [^198^Au]**3**-1 and [^198^Au]**3**-2 a typical absorption for AuNPs at 514 nm was observed, indicating stable AuNPs even 5 months after neutron activation ([^198^Au]**3**-3, Figure A9). The production of ~100 MBq [^198^Au]**3** showed stable AuNPs even at high activity concentration for at least 15 d by HPLC measurements (Figure A10).

### 2.3. Cell Experiments

#### 2.3.1. Determination of Target Avidities

**Figure A3:**
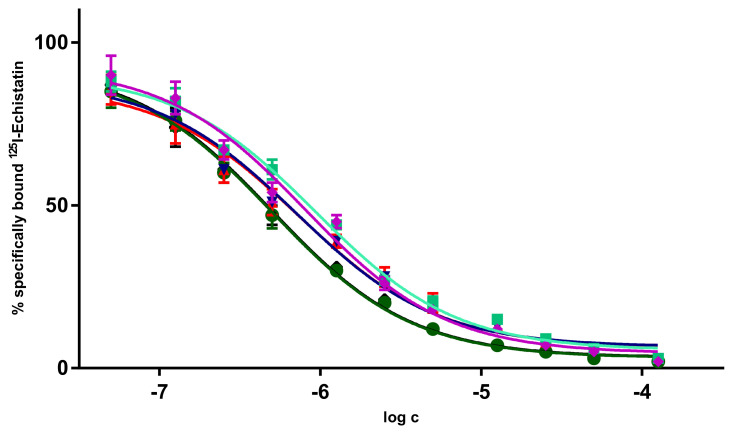
Binding curves of c(RGDfK). Different color means different experiment.

**Figure A4:**
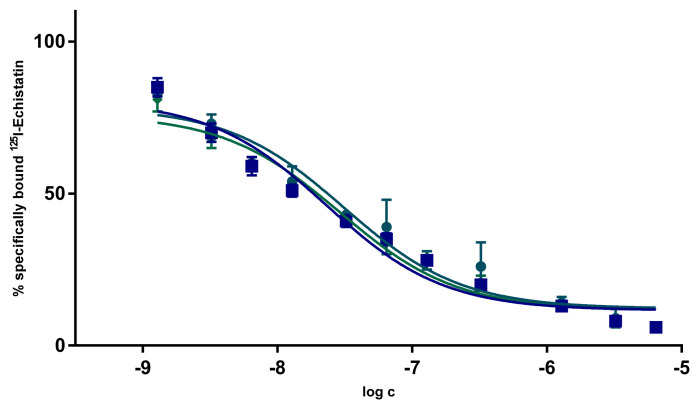
Binding curves of AuNPs **7a**. Different color means different experiment.

**Figure A5:**
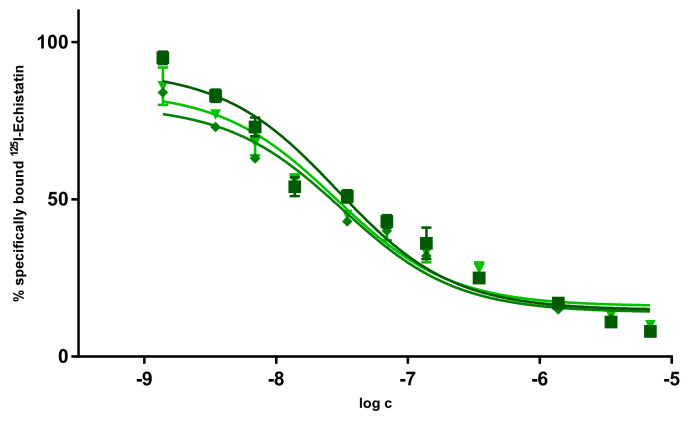
Binding curves of AuNPs **7b**. Different color means different experiment.

**Figure A6:**
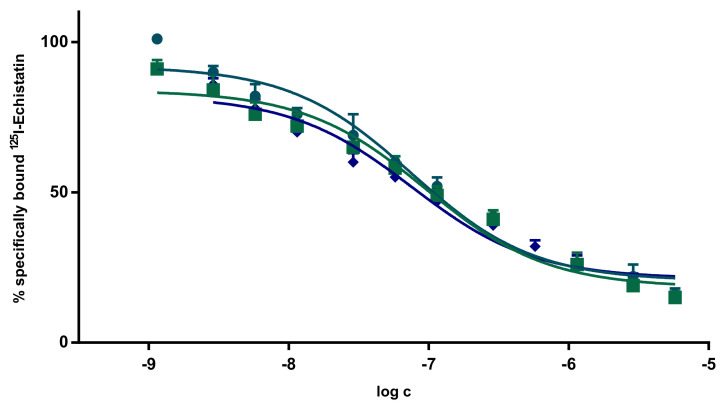
Binding curves of AuNPs **8a**. Different color means different experiment.

**Figure A7:**
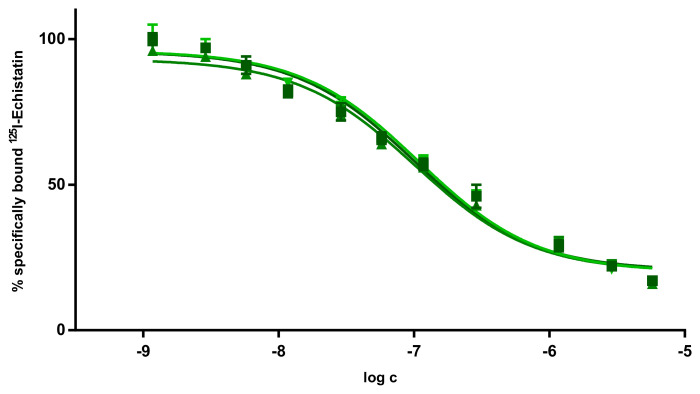
Binding curves of AuNPs **8b**. Different color means different experiment.

**Figure A8:**
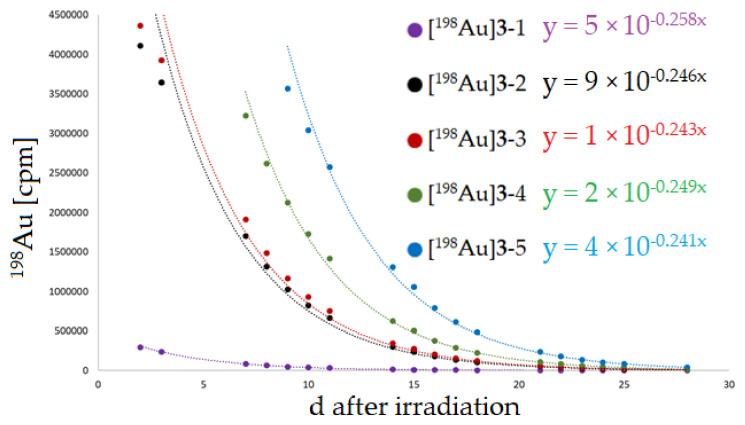
Decay curves of [^198^Au]**3** with different initial activities.

**Figure A9:**
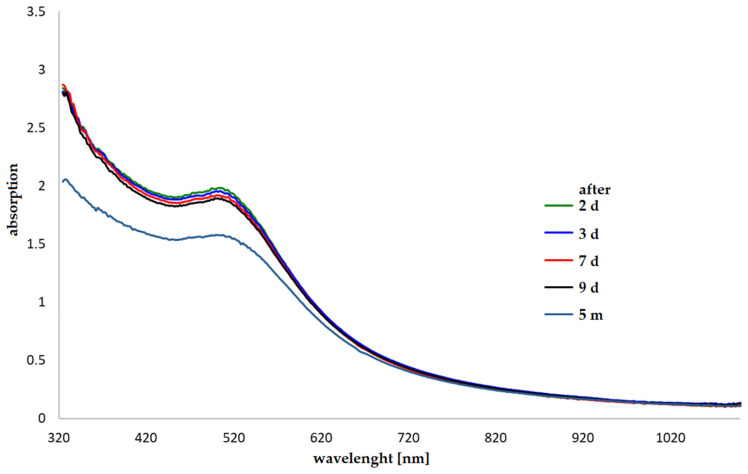
Representative absorption spectrum of [^198^Au]**3** at different time points after neutron activation with a typical absorption maximum for ultrasmall AuNPs at 514 nm indicating no aggregation of the particles.

Several different IC_50_ values for RGD derivatives have been reported in the literature, ranging from 0.1 nM up to 6.7 µM. The main reason for the observed differences is the assay method used to determine the IC_50_ values. IC_50_ values of 0.1–1 nM can be found for RGD peptides having been determined by ELISA assays [38] and IC_50_ values around 20 nM have been reported for solid-phase α_v_β_3_ binding assays for monomeric RGD derivatives [37]. Those IC_50_ values were derived by non-living experiments. However, cell experiments are closer to in vivo conditions. Therefore, for the AuNPs **7** and **8**, the α_v_β_3_ integrin-avidities were determined by competitive displacement experiments on α_v_β_3_-expressing U87MG cells using ^125^I-echistatin as the α_v_β_3_-specific radioligand and competitor. The RGD monomer c(RGDfK) was evaluated as an internal reference. The evaluation of RGD derivatives by displacement experiments yielded IC_50_ values comparable to those reported in the literature [34]. For the c(RGDfK) monomer, a mean IC_50_ value of 0.7 µM was determined (Table 3, Figure A3). The multi-RGD decoration on the surface of AuNPs **7a** and **7b** resulted in a lower mean IC_50_ value of 27.8 and 38.3 nM, respectively (Figure A4 and Figure A5). Mean IC_50_ values of 82.4 and 103.6 nM were found for AuNPs **8a** and **8b**, respectively (Figure A6 and Figure A7). It was observed that the higher the loading with α_v_β_3_-specific RGD peptide, the lower the IC_50_ values.

#### 2.3.2. Determination of Cell Survival

Colony formation assays were performed with [^198^Au]**3** with U87MG cells. For this proof-of-concept experiment, 5–10 Gy was chosen as the incubation dose. To achieve this dose, 1–2 MBq [^198^Au]**3** per well in a 24-well plate within a 96 h incubation period was calculated using Formula (1).

(1) $$D\left(A,t\right)=S\times \frac{A*T_{1/2}}{\ln\left(2\right)}\left[1-\exp\left(-\ln\left(2\right)\times \frac{t}{T_{1/2}}\right)\right]$$

Formula (1)—Calculation of dose to a cell monolayer at the bottom of a multi-well plate or Eppendorf tube for ^198^Au using Geant4-simulation [44]. *D*: energy dose, *S*: S-value, *A*: activity, *T*_1/2_: half-life of the radionuclide, *t*: irradiation time.

**Figure 4:**
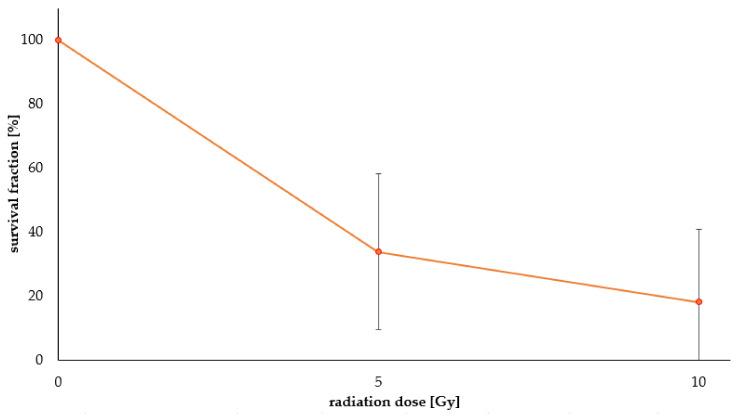
Survival fractions of the colony formation assays of 5 Gy [^198^Au]**3** and 10 Gy [^198^Au]**3**.

This dose corresponds to concentrations of [^198^Au]AuNPs of 0.515–0.939 µM, which is at least 10 times higher than the IC_50_ of AuNP-dithio-RGD **7** and **8**. It was observed that the survival fraction (sf) of the cells was significantly reduced for [^198^Au]**3** and that higher doses of 10 Gy (sf = 18.2%) were more effective in damaging the tumor cells than 5 Gy (sf = 33.9%) (Figure 4).

## 3. Discussion

c(RGDfK) is a highly potent and selective integrin α_v_β_3_ antagonist and therefore could disrupt cell viability by inhibiting angiogenesis [45]. Radiolabeled RGD derivatives can be used as tracers for tumor angiogenesis [46]. Multimerization leads to better tumor accumulation [47,48]. Therefore, AuNPs decorated with a multitude of c(RGDfK) motifs could lead to better tumor accumulation, which is important for therapy.

Methods for the preparation of [^198^Au]AuNPs are already known in the literature [11,29,32]. However, the synthesis starts with neutron activation of gold foil, which is then dissolved in aqua regia, followed by nanoparticle synthesis and further functionalizations with target-specific ligands. All these steps are performed with radioactive ^198^Au, resulting in higher dose accumulation for the personnel and more radioactive waste as the consequence. In this work, it was decided to first complete the synthesis of tumor-specific AuNPs with a high target avidity and high stability, and to perform neutron activation as the last step in order to reduce the personnel dose and enable a highly efficient synthesis pathway, which is mandatory for high clinical relevance. The challenge was to synthesize AuNPs that withstand neutron activation without aggregation and loss of the ligand shell.

Stable α_v_β_3_-specific AuNPs **7** and **8** were successfully synthesized with a better avidity compared to the monomeric peptide ligand c(RGDfK). During the irradiation experiments, it was observed that AuNPs containing monothiol ligands were unstable against neutron activation. However, all AuNP derivatives containing only dithiol ligands were stable against neutron activation even at the highest concentrations and irradiation times (~7.5 mg/mL within 2 h). It is known that sulfur can also be activated by neutrons via the ^32^S(*n*,*p*)^32^P reaction [49,50]. Presumably, once a sulfur atom is activated to ^32^P, it loses its covalent bond to the AuNP surface and a monothiol ligand is lost to the environment. In contrast, a dithiol ligand could remain bound to the surface even if a binding interaction is lost by activation of one of the sulfur atoms.

To determine the therapeutic influence of [^198^Au]AuNPs, cell survival was addressed by a colony formation assay. The activity and incubation time to reach relevant doses between 5 and 10 Gy were calculated for monolayer cell culture in 24-well plates (Formula (1)). To reach these doses of 5–10 Gy concentrations of a factor >10 times higher than the IC_50_ for AuNP-RGD **8a** and **8b** had to be used within 96 h of incubation. Therefore, cell viability should be considered to be very low when using such high concentrations of [^198^Au]**8** in cell survival experiments, as the antagonist RGD may interfere with angiogenesis and thus cell viability [45]. To circumvent this problem, future experiments should use higher activity concentrations (due to longer activation of AuNPs) or longer cell incubation with lower doses >5 Gy, when evaluating cell survival. However, in the proof-of-concept cell survival experiments, non-specific [^198^Au]**3** showed a significant effect on U87MG cells with a survival fraction as low as 18.2% at 10 Gy. Therefore, the combination of β^−^-emission from ^198^Au and the antagonistic effect of RGD could dramatically reduce the therapeutically relevant dose of applied [^198^Au]AuNP-RGDs.

## 4. Materials and Methods

**Figure A10:**
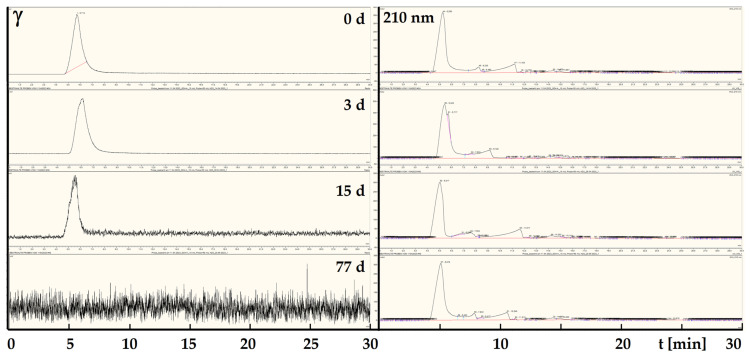
Representative (radio-)chromatogramms of [^198^Au]**3** showing no degradation or aggregation within 77 days. The [^198^Au]**3** *t*_R_ = 5.7 min. Ascorbic acid *t*_R_ = 11.6 min.

**Figure A11:**
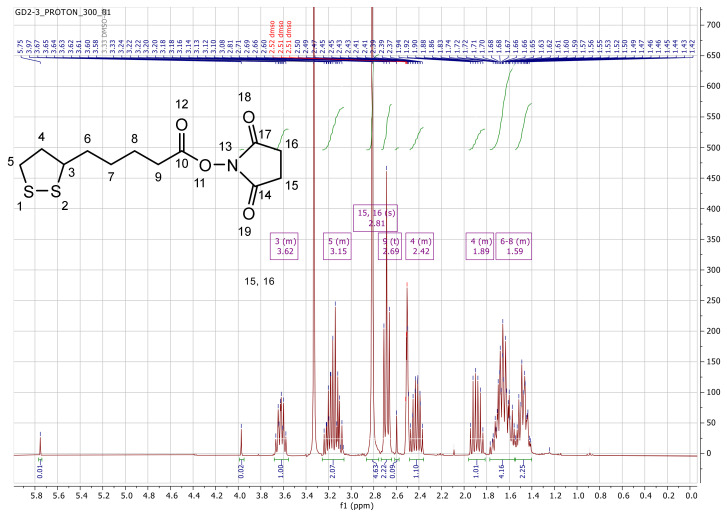
^1^H NMR spectrum of TA-NHS **1** in d_6_-DMSO.

**Figure A12:**
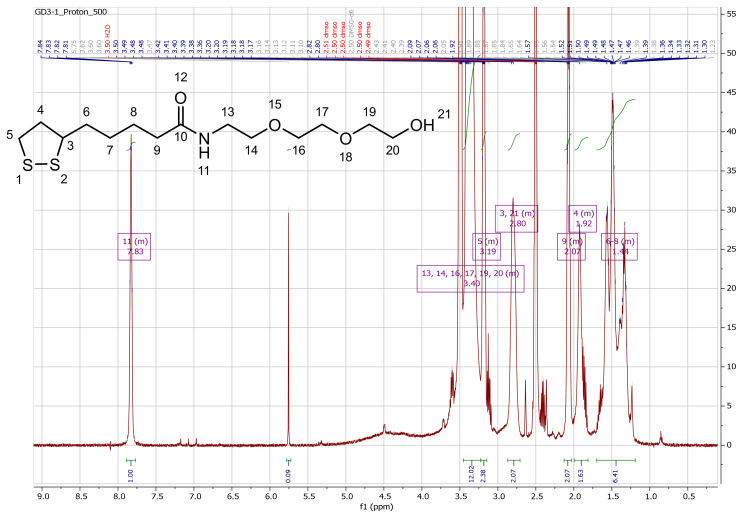
^1^H NMR spectrum of TA-PEG_3_-OH **2** in d_6_-DMSO.

**Figure A13:**
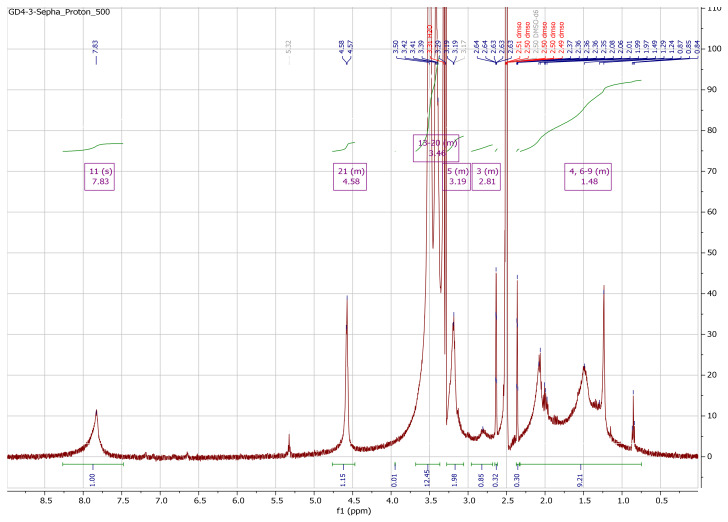
^1^H NMR spectrum of AuNP-dithio-PEG **3** in d_6_-DMSO.

**Figure A14:**
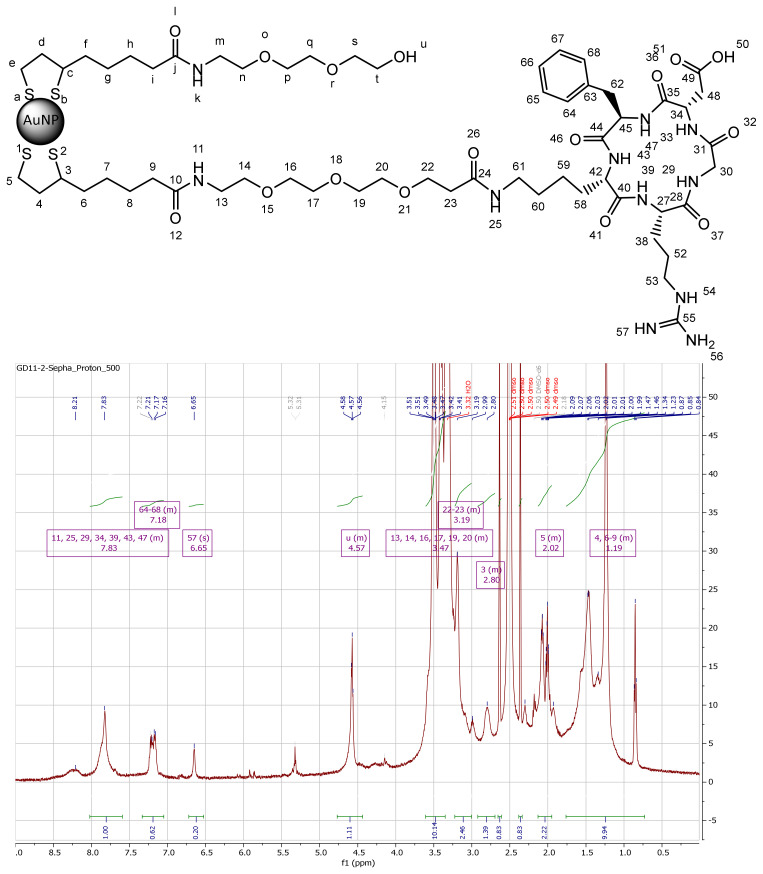
^1^H NMR spectrum of AuNP-dithio-RGD_high_ **8a** in d_6_-DMSO: characteristic signals of peptide-bond-NH (7.83 ppm), Phe-CH_arom_ (7.22–7.16 ppm), Arg-C=NH (6.65 ppm), and Asp-CH (5.32 ppm) can be found.

**Figure A15:**
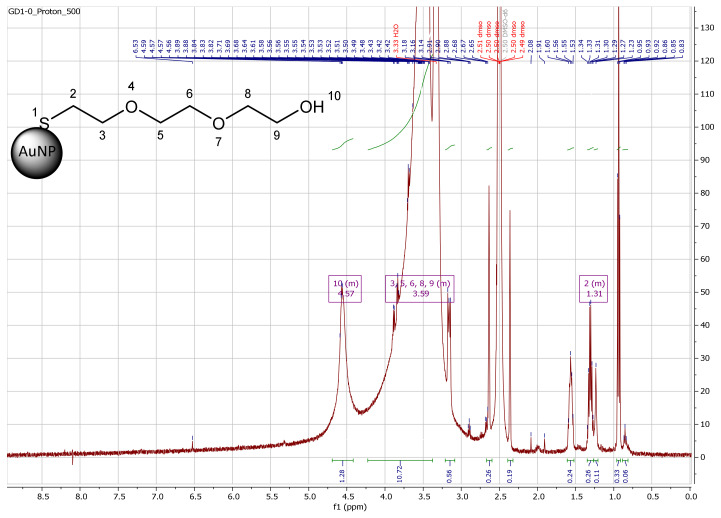
^1^H NMR spectrum of AuNP-thio-PEG **6** in d_6_-DMSO.

**Figure A16:**
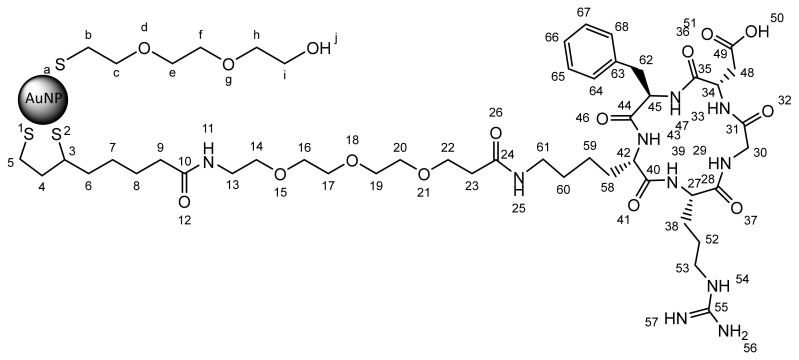

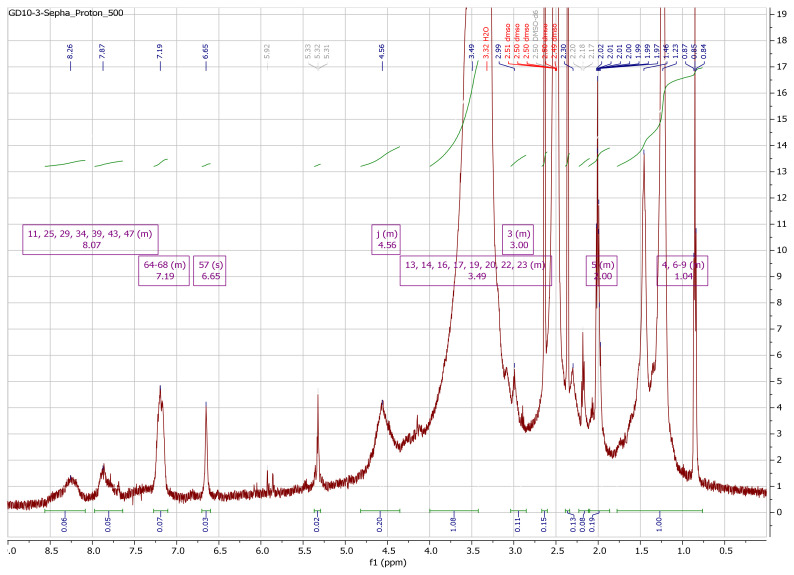
^1^H NMR spectrum of AuNP-thio-dithio-RGD_high_ **7a** in d_6_-DMSO: characteristic signals of peptide-bond-NH (7.87 ppm), Phe-CH_arom_ (7.19 ppm), Arg-C=NH (6.65 ppm), Arg-NH_2_ (5.92 ppm), and Asp-CH (5.32 ppm) can be found.

General procedures. All reagents and solvents were purchased from commercial suppliers (Sigma, Merck) and were used without further purification. NMR spectra were recorded on a 300 MHz Mercury Plus and a 500 MHz NMR System spectrometer (Varian, Palo Alto, CA, USA). Chemical shifts (*δ*) are given in ppm and are referenced to the residual solvent resonance signals relative to (CH_3_)_4_Si (^1^H, ^13^C). Mass spectra were obtained on a microflex MALDI-TOF mass spectrometer (Bruker Daltonics, Bremen, Germany) and HR-ESI-MS spectra on a LTQ FT Ultra Fourier Transform Ion Cyclotron Resonance spectrometer (Thermo Finnigan, Dreieich, Germany). When applicable, purity was determined by HPLC. The purity of all final compounds was 95% or higher. HPLC was performed on a Dionex UltiMate 3000 HPLC system (Thermo Scientific, Dreieich, Germany), equipped with a reverse phase column (Analytical: Merck Chromolith RP-18e; 100 × 4.6 mm plus a guard column 5 × 4.6 mm; semipreparative: Chromolith RP-18e; 100 × 10 mm plus a guard column 10 × 4.6 mm), and a UV-diode array detector (210 nm, 254 nm). The solvent system used was a gradient of acetonitrile:water (containing 0.1% TFA) (0–5 min: 0–100% MeCN) at a flow rate of 4 mL/min, unless otherwise stated. The purity and stability of AuNPs/[^198^Au]AuNPs were investigated by size exclusion HPLC using a PolySep™-SEC GFC-P 4000, LC column 300 × 7.8 mm, and a 35 mm PolySep guard column (Phenomenex, Aschaffenburg, Germany) with water (0.8 mL/min) as eluent (Figure A10). Purification of AuNPs was performed by dialysis (tubes with molecular weight cut-off of 14,000 g/mol, Visking, Roth, Karlsruhe, Germany) against distilled water and by size-exclusion chromatography using Sephadex G25 PD10 columns (Fisher Scientific, Schwerte, Germany) and distilled water as eluent. 

A brief description of the AuNP syntheses can be found in Appendix A.

Determination of the number of ligands on the surface of the AuNPs. The thermogravimetric analyses were performed using a Mettler Toledo TGA 2 STAR^e^ system. AuNPs (1–2 mg) were weighed into 70-µL-aluminum oxide crucibles (Mettler Toledo, Gießen, Germany) and heated from 25–750 °C (10 K/min) in a stream of N_2_ or CO_2_ (30 mL/min). The loading of the different AuNPs is shown in Table 1 and was calculated from the different mass losses, which increase as the AuNPs are functionalized. Therefore, the amount of different ligands per particle can be calculated according to the formula of Zhu et al. [42]. Since the nanoparticles have an average diameter of ~3 nm, the calculated amount of gold atoms is ~834 Au atoms per nanoparticle. This gives a molecular weight of an AuNP of 164,298 g/mol. Using TGA, the following ligand numbers were determined:

- The mass loss of the AuNP **6** was ~19.8%. This corresponds to ~250 PEG ligands on the AuNP surface. M~210 kDA.
- The mass loss of AuNP-RGD **7a** was ~24.8% and the RGD accounts for ~5% mass loss (~35 RGD ligands per AuNP). Therefore, the molar mass for AuNP-RGD_high_ **7a** was calculated to be ~239 kDa.
- Furthermore, the AuNP-RGD_low_ **7b** contained ~15 RGD ligands ~222 kDa.
- The mass loss of the AuNP **3** was ~33.27%. results in ~240 PEG ligands on the AuNP surface. M~246 kDa.
- The mass loss of AuNP-PEG-RGD_high_ **8a** was ~37.1% and the RGD accounts for ~4% mass loss (~24 RGD ligands per AuNP). Therefore, the molar mass for AuNP-RGD_high_ **8a** was calculated to be ~262 kDa.
- Furthermore, the AuNP-RGD_low_ **8b** contained ~18 RGD ligands ~257 kDa.

Avidity experiments. The α_v_β_3_-binding affinities of the respective RGD peptides and AuNPs were determined using in vitro competitive displacement experiments on U87MG tumor cells (HTB-14, ATCC^®^, Manassas, VA, USA). U87MG cells were harvested and resuspended in the binding buffer at a cell concentration of 2 × 10^6^/mL to reach 10^5^ cells per well.

A special binding buffer in sterile distilled water (Tris·HCl 25 mM, NaCl 150 mM, CaCl_2_ 1 mM, MgCl_2_ 0.5 mM, MnCl_2_ 1 mM, pH 7.4, BSA 0.5%) was used for incubation with 0.25–0.40 kBq/well ^125^I-Echistatin (81.4 GBq/μmol) as the α_v_β_3_ specific radioligand in the presence of increasing concentrations (0–100 μM) of competing c(RGDfK) peptide or c(RGDfK)-modified AuNPs (0–20 µM). IC_50_ values were obtained using GraphPad Prism v6.05 (nonlinear fit) software.

Neutron irradiation experiments. Production of [^198^Au]AuNPs by neutron activation of 0.05–15.5 mg AuNPs was performed in pneumatic transfer tube one for 1–2 h at 100 kW with a thermal neutron flux of 1.6 × 10^12^ cm^−2^ × s^−1^ in the TRIGA research reactor (Mainz, Germany). For calibration of the dose calibrator ISOMED 2010 (NUVIA Instruments, Dresden, Germany) 12.7 mg solid Au was irradiated for 1 h to produce 87 MBq (calculated) [^198^Au]Au with a measured dose rate of 57 µSv/h. 26 h later, the activity was measured with the dose calibrator, and 60 MBq was obtained (using the ^137^Cs-channel, 66 MBq calculated). In addition, the solid [^198^Au]Au (40 MBq) was carefully dissolved in 2 mL aqua regia at 50 °C within 15 min in order to find the correct calibration factors of the dose calibrator for different volumes in vials and syringes.

Irradiation of AuNPs was performed under optimized conditions in 2 mL 10% EtOH/H_2_O and 25 mg ascorbic acid as a stabilizer against radiolysis [51]. Theoretically, 10 mg of pure ^197^Au irradiated with a thermal neutron flux of 1.6 × 10^12^ cm^−2^ × s^−1^ would produce 48–96 MBq ^198^Au within 1–2 h of irradiation. In the experiment, neutron activation of 5.0 mg AuNPs **3** and **8** for 2 h produced 48 MBq [^198^Au]**3** (66.7% Au) and 50 MBq [^198^Au]**8** (62.9% Au). Neutron activation of 15.56 mg AuNP **3** for 2 h produced ~100 MBq [^198^Au]**3** (67% Au). The production of ^198^Au was confirmed by gamma spectroscopy, which found up to three gamma lines at 411 keV (95.6%), 676 keV (0.8%), and 1088 keV (0.2%).

Colony formation assay. Three days before the experiments, 150,000 cells were seeded in 24-well plates. U87MG cells were incubated for 96 h in the presence of the α_v_β_3_-specific or non-radioactive AuNPs or 1–2 MBq [^198^Au]AuNPs to achieve the calculated doses of 5–10 Gy. After incubation, the cell medium was removed, the cells were washed and harvested, and a colony formation assay was performed in triplicate for each irradiation point with 1000 cells per well in a 6-well plate. Colonies were cultured in cell medium for 28 days, then washed with 1 mL PBS, fixed with 2 mL 4% formaldehyde in PBS for 15 min, and incubated with 2 mL 0.5% crystal violet dye solution for 30 min. Afterward, colonies were washed with distilled water, dried, and counted by light microscopy. Colonies of more than 50 cells were considered viable, and the plating efficiency for each sample was estimated based on the initial number of cells seeded. Clonogenic cell survival was calculated as the relative plating efficiency of treated versus untreated samples. Triplicate samples were prepared for each treatment and experimental condition.

## 5. Conclusions

α_v_β_3_-specific RGD-containing AuNPs with a higher target avidity compared to α_v_β_3_-specific RGD were successfully synthesized. This proof-of-concept work should demonstrate, that activation of AuNPs with a ligand shell is possible without losing their organic shell and integrity. Irradiation experiments demonstrated the stability and consistency of [^198^Au]AuNPs with dithiol ligands compared to [^198^Au]AuNPs with monothiol ligands, which always aggregated at each applied concentration after neutron activation. In vitro experiments determine the therapeutic effect of [^198^Au]AuNPs by addressing the survival fraction of U87MG cells proved a significant influence on cell death. Therefore, the [^198^Au]AuNPs could serve as a tool for endoradiotherapy.

Further experiments to determine the therapeutic effects of [^198^Au]AuNPs in vivo by different modes of application (local vs. systemic) are currently underway.

## Figures and Tables

**Table 1:** Calculated number of ligands and resulting molecular mass of the AuNPs.

Probe	Description	Number of Ligands	Molecular Mass [kDa]
**6**	AuNP-PEG	250 × thio-PEG	210
**7a**	AuNP-PEG-RGD_high_	152 × thio-PEG, 35 × **5**	239
**7b**	AuNP-PEG-RGD_low_	196 × thio-PEG, 15 × **5**	222
**3**	AuNP-dithio-PEG	240 × **3**	246
**8a**	AuNP-dithio-PEG-RGD_high_	218 × **3**, 24 × **5**	262
**8b**	AuNP-dithio-PEG-RGD_low_	220 × **3**, 18 × **5**	257

**Table 2:** Summary of neutron activation of various AuNPs, mass, and calculated half-life.

Probe	Weight [mg]	DR ^1^ [µSv/h] in 1 cm/30 cm after				Precipitation Observed	t_1/2_ [d] (Calc.)
		5 min	10 min	30 min	60 min		
[^198^Au]**3**-1	0.05	55/4	35/3	25/3	25/3	no	2.6866
[^198^Au]**3**-2	0.50	125/5	125/5	115/4	100/4	no	2.8177
[^198^Au]**3**-3	0.75	170/5	160/5	155/4	150/4	no	2.8525
[^198^Au]**3**-4	1.00	250/6	215/5	210/5	212/5	no	2.7837
[^198^Au]**3**-5	2.00	500/8	450/8	420/8	410/8	no	2.8761
[^198^Au]**6**-1	0.05	76/3.7	n.d. ^2^	n.d. ^2^	n.d. ^2^	yes	n.d. ^2^
[^198^Au]**6**-2	0.50	150/n.d. ^2^	15/6	n.d. ^2^/5.3	n.d. ^2^	yes	n.d. ^2^
[^198^Au]**6**-3	5.06	n.d. ^2^	n.d. ^2^	1000/n.d. ^2^	n.d. ^2^	yes	n.d. ^2^
[^198^Au]**8**a-1	0.05	n.d. ^2^	n.d. ^2^	15/5	n.d. ^2^	no	n.d. ^2^

^1^ DR: dose rate; ^2^ n.d.: not determined.

**Table 3:** Avidity experiments.

Probe	Description	IC_50_ [nM]
c(RGDfK)	α_v_β_3_ antagonist	700.4 ± 155.9
**7a**	AuNP-PEG-RGD_high_	27.8 ± 3.4
**7b**	AuNP-PEG-RGD_low_	38.3 ± 11.9
**8a**	AuNP-dithio-PEG-RGD_high_	82.4 ± 9.2
**8b**	AuNP-dithio-PEG-RGD_low_	103.6 ± 3.5

## Data Availability

All data can be referred to on request to the corresponding author.

## Appendix A

### Appendix A.1. Organic Syntheses

**c(RGDfK)** [52]

The peptide c(RGDfK) c(Arg-Gly-Asp-D-Phe-Lys) was synthesized according to standard protocols by solid-phase peptide synthesis on solid support using the Fmoc-strategy on H-Asp(*t*Bu)-2-chlortrityl resin (loading 0.73 mmol/g, 137 mg, 0.1 mmol). For amino acid conjugation, HBTU (*N*,*N*,*N′*,*N′*-tetramethyl-*O*-(1*H*-benzotriazol-1-yl)uronium hexafluorophosphate) (3.9 eq., 0.39 mmol, 148.7 mg), Fmoc-protected amino acids (4.0 eq., 0.4 mmol) and DIPEA (4.0 eq., 0.4 mmol, 68 µL) were used in DMF as solvent. Each amino acid was coupled for 45 min. After coupling of the last amino acid and Fmoc-removal with 50% of piperidine solution in DMF, the linear protected peptide was cleaved from the resin using 1% TFA in CH_2_Cl_2_. After removal of the volatile components of the mixture, the crude intermediate was isolated and dissolved in dry DMF (85 mL). After addition of DIPEA (3.5 eq., 0.35 mmol, 59.5 µL) the solution was cooled to 0 °C and DPPA (1.25 eq., 0.125 mmol, 26.9 µL) was added. The reaction was allowed to warm to ambient temperature and stirred for 3 days until the cyclization was complete. The volatile components of the mixture were evaporated under reduced pressure, and the residue was treated with a mixture of TFA/TIS 97.5:2.5 for 3 h to completely deprotect the peptide. The crude product was precipitated in cold diethyl ether and washed twice with diethyl ether and dried under reduced pressure. The product was purified by semi-preparative HPLC and lyophilized to give a colorless solid (yield: 88.79%, 53.6 mg, 0.089 mmol). HR-ESI-MS (*m*/*z*) for [M + H]^+^ (calculated): 604.3 (604.3). MALDI-MS (*m*/*z*) for [M]^+^ (calculated): 603.7 (603.3).

**TA-NHS 1** [53]

Thioctic acid (TA) (1 eq., 2.425 mmol, 0.50 g) was dissolved in acetone (12.5 mL) under an argon atmosphere. *N*,*N*-Disuccinimidyl carbonate (1.25 eq., 3.031 mmol, 0.78 g) and DIPEA (1.25 eq., 3.031 mmol, 0.5 mL) were added and the mixture was stirred overnight at room temperature. After removal of acetone under reduced pressure, the residue was dissolved in 10 mL of water and DCM (1:1). The aqueous phase was removed and the organic phase was washed twice with 4 mL water. After drying the organic phase with MgSO_4,_ the solvent, DCM, was removed and TA-NHS was obtained as a yellowish solid. (78.01%, 574 mg, 1.89 mmol). ^1^H-NMR (300 MHz, DMSO-d_6_) *δ* = 3.67–3.58 (m, 1H, *H*-3), 3.24–3.08 (m, 2H, *H*-5), 2.81 (s, 4H, *H*-15, *H*-16), 2.69 (t, ^3^*J* = 7.2 Hz, 2H, *H*-9), 2.47–2.37 (m, 1H, *H*-4), 1.94–1.83 (m, 1H, *H*-4), 1.77–1.42 ppm (m, 6H, *H*-6, *H*-7, *H*-8).

**TA-PEG_3_-OH 2**

TA-NHS dissolved in 3 mL DCM (1.2 eq., 0.96 mmol, 0.29 g) was added to a solution of H_2_N-PEG_3_-OH (1 eq., 0.80 mmol, 0.12 g) in DCM (1 mL). After the addition of DIPEA (4 eq., 3.2 mmol, 545 µL) was added and the mixture was stirred overnight at ambient temperature. The solvent was removed under reduced pressure, and the crude product was purified by semi-preparative HPLC. Finally, the product TA-NH-PEG_3_-OH was isolated as yellowish viscous liquid (yield: 65.04%, 180 mg, 0.533 mol). ^1^H-NMR (500 MHz, DMSO-d_6_) *δ* = 7.89–7.77 (m, 1H, *H*-11), 3.43–3.37 (m, 12H, *H*-13, *H*-14, *H*-16, *H*-17, *H*-19, *H*-20), 3.22–3.15 (m, 2H, *H*-5), 2.87–2.71 (m, 2H, *H*-3, *H*-21), 2.13–2.03 (m, 2H, *H*-9), 1.99–1.81 (m, 2H, *H*-4), 1.60–1.21 ppm (m, 6H, *H*-6, *H*-7, *H*-8). HR-ESI-MS (*m*/*z*) for [M + H]^+^ (calculated): 338.1 (338. 1), [M + Na]^+^ (calculated): 360.1 (360.1). MALDI-MS (*m*/*z*) for [M]^+^ (calculated): 337.3 (337.1), [M + H]^+^ (calculated): 338.1 (338. 1).

**TA-PEG_4_-COOH 4**

TA-NHS dissolved in 2 mL DCM (1.1 eq., 0.88 mmol, 0.27 g) was added to a suspension of H_2_N-PEG_4_-COOH (1 eq., 0.80 mmol, 0.12 g) in DMF (2 mL). DIPEA (4 eq., 3.2 mmol, 545 µL) was added and the mixture was stirred overnight at ambient temperature. The solvent was removed under reduced pressure, and the crude product was purified by semi-preparative HPLC. After lyophilization the product TA-NH-PEG_4_-COOH was obtained as colorless solid (yield: 48.92%, 160.3 mg, 0.39 mmol). HR-ESI-MS (*m*/*z*) for [M + H]^+^ (calculated): 410.1 (410.1), [M + Na]^+^ (calculated): 432.1 (432.1). MALDI-MS (*m*/*z*) for [M]^+^ (calculated): 409.4 (409.1), [M + K]^+^ (calculated): 447.4 (448.1).

**TA-PEG_4_-c(RGDfK) 5**

TA-NH-PEG_4_-COOH dissolved in 0.5 mL of DMF (1.1 eq., 0.055 mmol, 22.4 mg) was added to a solution of PyBOP (1.9 eq., 0.094 mmol, 49.1 g) in 0.5 mL of DMF. DIPEA (3 eq., 0.149 mmol, 26 µL) was added and the mixture was stirred at ambient temperature until the reaction was completed. DMF was then removed under reduced pressure, and the crude product was purified by semi-preparative HPLC. After lyophilization the product TA-NH-PEG_4_-c(RGDfK) was obtained as colorless solid (yield: 28.91%, 14.3 mg, 0.014 mmol). HR-ESI-MS (*m*/*z*) for [M + H]^+^ (calculated): 995.4 (995.4). MALDI-MS (*m*/*z*) for [M]^+^ (calculated): 994.8 (994.4), [M + K]^+^ (calculated): 1033.7 (1033.4).

**AuNP-dithio-PEG_3_-OH 3** [40]

Hydrogen tetrachloroaurate (1 eq., 0.525 mmol, 207 mg) was dissolved in 205 mL MeOH to give a bright yellow solution and under stirring a solution of TA-NH-PEG_3_-OH dissolved in 205 mL of MeOH was added and stirred for 2 h until the reaction color became nearly colorless. A solution of sodium borohydride dissolved in 20.5 mL of water was quickly added to the reaction. The solution immediately turned black. The reaction mixture was stirred overnight, MeOH was removed under reduced pressure, and the residue was redissolved in 9 mL of tracepure water and dialyzed in distilled water for 4 days. After lyophilisation TA-AuNP was obtained as black powder. (39.47%, 180.7 mg).

**AuNP-thio-PEG 6** [6]

General procedure for the preparation of PEGylated AuNPs: Briefly, hydrogen tetrachloroaurate(III) trihydrate (1 eq., 0.4 mmol, 157.5 mg, ≥99.9% trace metal basis) was dissolved in 12.5 mL of trace pure water resulting in a bright yellow solution, and then extracted by mixing with 125 mL of a tetraoctylammonium bromide (TOABr, 1.2 eq., 0.48 mmol, 263 mg) toluene solution. The contents were stirred vigorously for 20 min at room temperature to facilitate the phase transfer of the Au(III) into the toluene layer, which resulted in the organic layer turning to a dark orange color and the aqueous layer becoming clear colorless. After the phase transfer was complete, the aqueous layer was removed. The organic layer was dried with MgSO_4_ and filtered to remove excess of water. The solution was cooled to 0 °C in an ice bath. Freshly-prepared HO-PEG_3_-thiol (3 eq., 1.2 mmol, 199 mg) in 6.3 mL of dichloromethane was added and stirred until the orange solution turned to colorless (~1 h). A fresh solution of tetrabutylammonium borohydride (TBABH) (10 eq., 4.0 mmol, 1.03 g) in 6.3 mL dichloromethane was then added to the rapidly stirring toluene solution over 5 s. The solution immediately turned black. The PEG-AuNP began to precipitate from the toluene after 1 h. After stirring the mixture for 16 h from 0 °C to 20 °C, 6.3 mL of trace pure water was added under slow stirring to extract the PEGylated AuNPs for 120 min. The organic layer was decanted and the aqueous layer was washed alternately with 3 × 13 mL toluene/1.3 mL MeCN and 3 × 13 mL toluene/1.3 mL isopropanol. The black aqueous layer was transferred to a Visking cellulose dialysis tube (molecular cut-off 14,000 Da) with 3 × 6.3 mL trace pure water and dialyzed in 3 × 10 L of distilled water for 1 h, 2.5 h and 16 h. The AuNPs were lyophilized to yield 69.5 mg (25.10%) of black powder. ^1^H-NMR (500 MHz, DMSO-d6) *δ* = 4.67–4.48 (m, 1H, H-10), 3.93–3.38 (m, 10H, H-3, H-5, H-6, H-8, H-9), 1.36–1.25 ppm (m, 2H, H-2).

**AuNP-PEG-RGDs by ligand exchange**

General procedure for the preparation of RGD-decorated AuNPs: Briefly, the functionalization of AuNPs **3** and **6** was performed by a place-exchange reaction with TA-PEG_4_-c(RGDfK) **5**. TA-PEG_4_-c(RGDfK) **5** was dissolved in a mixture trace pure H_2_O:MeOH (1:1) and was added to AuNPs **3** or **6** in 2 mL trace pure H_2_O and stirred overnight. Purification of AuNPs was performed in two steps: First, the AuNP solution was transferred into a Visking cellulose dialysis tube (molecular cut-off 14,000 Da) with 3 × 1 mL trace pure water and dialyzed in distilled water for 4 days, and second, the AuNP solution was eluted by size-exclusion chromatography using Sephadex G25 PD10 columns and distilled water. The AuNPs were then lyophilized and obtained as a black powder.

**AuNP-thio-PEG_3_-dithio-PEG_4_-RGD_high_ 7a**

TA-PEG_4_-c(RGDfK) **5** (8 mg, 8.04 µmol) was dissolved in 0.4 mL of trace pure H_2_O:MeOH (1:1) and was added to AuNP **6** (20 mg) in 2 mL of trace pure H_2_O and stirred overnight. After purification and lyophilization 13.1 mg (57.9%) of **7a** was obtained as black powder. ^1^H-NMR (500 MHz, DMSO-d_6_) *δ* = 8.43–7.66 (m, 7H, *H*-11, *H* -25, *H*-29, *H*-34, *H*-39, *H*-43, *H*-47), 7.21–7.18 (m, 5H, *H*-64, *H*-65, *H*-66, *H*-67, *H*-68), 6.65 (s, 2H, *H*-57), 4.58–4.54 (m, 1H, *H*-j), 4.00–3.42 (m, 16H, *H*-13, *H*-14, *H*-16, *H*-17, *H*-19, *H*-20, *H*-22, *H*-23), 3.02–2.92 (m, 1H, *H*-3), 2.04–1.86 (m, 2H, *H*-5), 1.78–0.76 ppm (m, 10H, *H*-4, *H*-6, *H*-7, *H*-8, *H*-9).

**AuNP-thio-PEG_3_-dithio-PEG_4_-RGD_low_ 7b**

TA-PEG_4_-c(RGDfK) **5** (4 mg, 4.02 µmol) was dissolved in 0.4 mL of trace pure H_2_O:MeOH (1:1) and was added to AuNP **6** (20 mg) in 2 mL of trace pure H_2_O and stirred overnight. After purification and lyophilization 14.4 mg (68.4%) of **7b** was obtained as black powder.

**AuNP-dithio-PEG-RGD_high_ 8a**

TA-PEG_4_-c(RGDfK) **5** (8 mg, 8.04 µmol) was dissolved in 0.4 mL of trace pure H_2_O:MeOH (1:1) and was added to AuNP **3** (20 mg) in 2 mL of trace pure H_2_O and stirred overnight. After purification and lyophilization 20.8 (97.5%) of **8a** was obtained as black powder. ^1^H-NMR (500 MHz, DMSO-d_6_) *δ* = 8.02–7.59 (m, 7H, *H*-11, *H* -25, *H*-29, *H*-34, *H*-39, *H*-43, *H*-47), 7.22–7.16 (m, 5H, *H*-64, *H*-65, *H*-66, *H*-67, *H*-68), 6.65 (s, 2H, *H*-57), 4.77–4.43 (m, 1H, *H*-u), 3.61–3.35 (m, 10H, *H*-13, *H*-14, *H*-16, *H*-17, *H*-19, *H*-20), 3.22–3.00 (m, 4H, *H*-22, *H*-23), 2.92–2.69 (m, 1H, *H*-3), 2.13–1.95 (m, 2H, *H*-5), 1.76–0.73 ppm (m, 10H, *H*-4, *H*-6, *H*-7, *H*-8, *H*-9).

**AuNP-dithio-PEG-RGD_low_ 8b**

TA-PEG_4_-c(RGDfK) **5** (4 mg, 4.02 µmol) was dissolved in 0.4 mL of trace pure H_2_O:MeOH (1:1) and was added to AuNP **3** (20 mg) in 2 mL of trace pure H_2_O and stirred overnight. After purification and lyophilization 19.7 mg (95.1%) of **8b** was obtained as black powder.

### Appendix A.2. Electron Microscopy

AuNP samples were diluted at will in deionized water (fade red solution), particles were adsorbed onto glow-discharged carbon-coated EM grids and directly observed by TEM (Zeiss EM912, Carl Zeiss Oberkochen, Germany). Images were digitally captured with a CCD camera (Sharp eye, TRS, Moorenweiss, Germany). Particle number and size were measured using the FIJI software (v1.50e).

### Appendix A.3. Determination of Avidity of Non-Radioactive α_v_β_3_-Specific AuNPs

The α_v_β_3_-binding affinities of the respective RGD peptides and AuNPs were determined by in vitro competitive displacement experiments with U87MG tumor cells.

### Appendix A.4. Determination of Half-Life of [^198^Au]***3***

The half-life of [^198^Au]**3** was determined for 0.025–1.0 mg/mL by measuring five different probes for 28 days with a gamma counter (2470 WIZARD^2^, Perkin Elmer). A half-life of 2.80 ± 0.07 days was found (real: 2.69 days). Therefore, the half-life found in this experiment deviated by 4% from the real value (Figure A8).

### Appendix A.5. Determination of Stability of [^198^Au]AuNPs

#### Appendix A.5.1. UV/Vis Measurements

UV/Vis measurements were performed using an BioSpektrometer Kinetic (Eppendorf, Hamburg, Germany). The absorbance of [^198^Au]**3** at a concentration of 375 µg/mL was measured for up to 5 months (Figure A9) to estimate the particle size and stability. The absorption at surface plasmon resonance (maximum, 514 nm) divided by the absorption at 450 nm (minimum) gives a factor that can be compared with tables from the literature [54].

#### Appendix A.5.2. HPLC Measurements

10 µL of [^198^Au]**3** (7.75 mg/mL, 50 MBq/mL) was diluted with 50 µL H_2_O. 10 µL of the diluted solution was injected into an HPLC equipped with a Sephadex column. Measurements were performed up to 77 days after irradiation (Figure A10).
